# Sense of Purpose Promotes Resilience to Cognitive Deficits Attributable to Depressive Symptoms

**DOI:** 10.3389/fpsyg.2021.698109

**Published:** 2021-06-25

**Authors:** Nathan A. Lewis, Patrick L. Hill

**Affiliations:** ^1^Department of Psychology, University of Victoria, Victoria, BC, Canada; ^2^Institute on Aging and Lifelong Health, University of Victoria, Victoria, BC, Canada; ^3^Department of Psychological and Brain Sciences, Washington University in St. Louis, St. Louis, MO, United States

**Keywords:** depression, cognitive functioning, purpose in life, older adulthood, longitudinal studies

## Abstract

**Objective:**

Individuals higher in depressive symptoms commonly present with neuropsychological deficits including poorer memory performance. Sense of purpose in life has been shown to promote resilience to cognitive impairment in older adulthood, but it is unclear whether it may also protect against cognitive deficits associated with higher depressive symptoms.

**Method:**

Cognitive functioning among 4599 older American adults (*M*_*age*_ = 74.33 years, range = 65–104 years, 56.84% female) was examined across a 12-year follow-up period. Depressive symptomatology was assessed at each wave using the 8-item Center for Epidemiologic Studies Depression Scale. Multilevel models assessed the influence of depressive symptoms and the interaction with sense of purpose in life on changes in memory performance and mental status.

**Results:**

Higher depressive symptoms were associated with poorer memory performance at baseline, but did not predict rate of change over time. A negative interaction was observed between sense of purpose in life and depressive symptoms such that individuals higher in purpose experienced a less negative association between depressive symptoms and baseline memory performance. No significant interaction of sense of purpose and depressive symptoms was observed on mental status.

**Conclusion:**

Having a sense of purpose in life may help protect older adults from memory deficits associated with higher depressive symptoms. The present findings underscore the potential for sense of purpose to promote cognitive reserve in older adulthood, allowing individuals to maintain cognitive performance in the face of accruing neurological insults.

## Introduction

Multiple theoretical frameworks and a large empirical literature now supports the claim that sense of purpose is “a psychological resource for aging well” (Windsor et al., 2015, p. 975). Purpose in life is central to the Ryff model of psychological well-being in adulthood (Ryff, 1989, 2014), which describes it with respect to having a direction in life toward goal-directed activities. Moreover, theories of successful aging point to the value of productive life engagement (Rowe and Kahn, 1997), viewed by some as the defining characteristic of having a purpose in life (Scheier et al., 2006).

Although sense of purpose appears valuable for myriad reasons (Pfund and Hill, 2018; Pfund and Lewis, 2020), a burgeoning literature has pointed to its value for cognitive resilience in later life. In one study (Windsor et al., 2015), Australian older adults with a higher sense of purpose outperformed peers on measures of memory and processing speed. Moreover, the study found that purposeful older adults experienced reduced rates of decline for processing speed over time. Work with the MIDUS study in the United States has found similar results among middle-to-older adults; namely, sense of purpose was associated with better memory and executive functioning scores cross-sectionally (Lewis et al., 2017), and that changes in purpose were correlated with changes in memory skills (Dewitte et al., 2020).

Moving beyond normative cognitive resilience, sense of purpose also may protect against non-normative cognitive decline. For instance, one study found sense of purpose longitudinally predicted later risk for Alzheimer’s disease (Boyle et al., 2010). Moreover, sense of purpose was shown to moderate the association between the physiological markers for decline and diagnosis in that sample (Boyle et al., 2012) suggesting that having a sense of purpose may reduce the risk for decline posed by neurological factors. This work thus takes an interesting spin on the role of purpose in promoting healthy cognitive aging. Most studies in the area have discussed potential pathways by which sense of purpose may promote cognitive functioning (e.g., Windsor et al., 2015; Lewis et al., 2017; Dewitte et al., 2020), alerting researchers to consider whether purpose matters because it promotes physical health, emotional well-being, and activity engagement.

Instead, this work on Alzheimer’s risk points to an alternative rationale, namely that having a sense of purpose may mitigate risk for cognitive concerns. Such a proposition aligns with past work linking sense of purpose to greater resilience in later life (Nygren et al., 2005), insofar that it can provide a resource against adversity or obstacles. Multiple studies find that purposeful individuals report greater hope in their ability to navigate obstacles in life (Bronk et al., 2009; Burrow et al., 2010). However, the interpretation from this work is typically with respect to physical or perceived obstacles to goal pursuit, rather than for instance, psychological or physiological concerns. Some evidence toward the latter comes from work showing that sense of purpose may mitigate the impact of daily stressors on emotional and physical well-being (Hill et al., 2018). Given that stress may play a role in cognitive decline (e.g., Dickinson et al., 2011), the cumulative story from this work is that sense of purpose could play a role on cognitive ability by mitigating psychosocial risk factors. However, to this point, work has yet to investigate this purpose-as-mitigator account with non-neurological risk factors.

The current study investigated this claim with respect to whether sense of purpose serves to mitigate the risk associated with depressive symptoms. Several studies have demonstrated that depressive symptoms longitudinally predict cognitive decline, when measured using laboratory-based markers of cognitive performance (Yaffe et al., 1999; Wilson et al., 2004; Dotson et al., 2008), measures of mental status (Chodosh et al., 2007), and risk for non-normative decline (Wilson et al., 2002; Ownby et al., 2006). Even when longitudinal associations are not always found, studies have found depressive symptoms are linked with lower initial cognitive status (Ganguli et al., 2006).

When considering potential behavioral and psychological explanations for these findings, they elucidate the potential reasons why sense of purpose could help mitigate risks. To start, individuals with depressive symptoms are likely to have reduced interest in activity engagement, a protective factor that has been positively linked cross-sectionally and longitudinally to sense of purpose in older adulthood (Lewis and Hill, 2020). Furthermore, depressive symptoms include having poor quality sleep (Radloff, 1977), and sense of purpose prospectively predicts a reduced risk for sleep disturbance (Kim et al., 2015). Finally, sense of purpose has been linked to reduced risk for social anxiety (Kashdan and McKnight, 2013), and a greater likelihood for supportive relationships (Weston et al., 2020), which may counteract the disinterest in social engagement associated with depression. Given that activity engagement, social involvement, and sleep all promote healthy cognitive aging (Smith, 2016), sense of purpose appears to be a likely candidate for moderating the risk for cognitive deficits associated with depressive symptoms.

### Current Study

The current study tested this claim using longitudinal data from older adult participants in the Health and Retirement Study (HRS). Across occasions spanning 12 years, participants completed assessments for memory recall and mental status, and measures of sense of purpose and depressive symptoms. Previous work has demonstrated that sense of purpose predicts risk for non-normative cognitive decline when accounting for depressive symptoms (Boyle et al., 2010), however no studies to our knowledge have investigated whether these variables interact to predict cognitive ability in older adulthood. We tested whether sense of purpose would moderate cross-sectional and longitudinal associations between depressive symptoms and cognition, both with respect to recall and mental status.

## Method

### Participants

The current study utilized data from the HRS (Sonnega et al., 2014), a biennial nationally representative longitudinal study of American adults over the age of 50. In 2006, the HRS introduced a psychosocial questionnaire including measures of sense of purpose in life administered to a randomly selected half of the sample (Smith et al., 2017). This questionnaire was repeated at 4-year intervals, resulting in up to four repeated assessments of sense of purpose across a 12-year span between 2006 and the most recent 2018 wave. For the current study, eligible participants included those with available data for sense of purpose in life, depressive symptoms, and cognitive functioning who were aged 65 or older at the 2006 baseline wave. The resulting sample included 4599 participants.

### Materials

#### Cognitive Functioning

Cognition in the HRS was assessed using the Telephone Interview for Cognitive Status (TICS), a brief, non-clinical assessment designed to be administered *via* telephone. Based on previous validation of the cognitive measures in HRS (McArdle et al., 2007), cognition was subdivided into two domains: word recall and mental status. Word recall is a composite measure of immediate and delayed word recall memory tasks in which participants are provided with a list of 10 unrelated nouns to be recalled immediately after presentation and again after a 30-min delay. Scores on this composite measure range from 0 to 20. Mental status is a summary variable representing performance on tasks of numeracy and perceptual orientation including serial 7 and backwards counting tasks, as well as date (day, month, year, and day of week), object (e.g., “What tool do you usually use to cut paper?”), and United States president naming. Scores on the composite mental status measure range from 0 to 15.

#### Depressive Symptomatology

Depressive symptoms were assessed at each wave using the 8-item Center for Epidemiologic Studies Depression Scale, which has been previously validated in large survey data of older adults (Karim et al., 2015). Participants were asked to report whether they had experienced (yes = 1, no = 0) eight specific symptoms for much of the previous week (felt depressed, lonely, sad, unmotivated, happy, everything was an effort, enjoyed life, and had restless sleep). Positive items (e.g., felt happy) were reverse-scored and the sum was taken to create a score ranging from 0 to 8, with higher values indicating more depressive symptoms.

#### Sense of Purpose in Life

Sense of purpose in life was measured using the 7-item version of the Ryff Psychological Well-Being Scale (Ryff, 1989). This measure included items such as “I live life one day at a time and don’t really think about the future,” “I am an active person in carrying out the plans I set for myself,” and “I have a sense of direction and purpose in my life.” Participants were asked to rate on a six-point scale, from strongly disagree (1) to strongly agree (6), the extent to which such statements apply to them. The mean of these items was calculated to create a sense of purpose score for each wave.

### Statistical Analyses

Multilevel modeling (Raudenbush and Bryk, 2002) was used to examine changes in word recall and mental status across the 12-year follow-up. Multilevel modeling with maximum likelihood estimation was chosen as it is robust to missing data issues common in longitudinal studies and it permits the examination of both within-person and between-person dynamics on change in cognitive performance over time (Hoffman and Stawski, 2009). We utilized two different centering techniques to account for within- and between-person sources of variation in sense of purpose and depressive symptoms. At the within-person level (Level-1), sense of purpose and depressive symptoms were person-mean centered to reflect fluctuations over time in individuals’ scores on these variables relative to their own typical levels. At the between-person level (Level-2), sense of purpose and depressive symptoms were centered on the overall sample mean to account for consistent differences between individuals across the follow-up period. In this regard, the models for the present study can be expressed as:

Level 1:

Cognition_*ti*_ = β*_0__*i*_* + β*_1__*i*_*(Time) + β*_2__*i*_*(Purpose_*WP*_) + β*_3__*i*_*(CESD_*WP*_) + *e*_*ti*_

Level 2:

β*_0__*i*_* = γ*_00_* + γ*_01_*(Age) + γ*_02_*(Sex) + γ*_03_*(Education) + γ*_04_*(Purpose_*BP*_) + γ*_05_*(CESD_*BP*_) + γ*_06_*(Purpose × CESD) + μ*_0__*i*,_*

β*_1__*i*_* = γ_10_ + γ*_11_*(Age) + γ*_12_*(Sex) + γ*_13_*(Education) + γ*_14_*(Purpose_*BP*_) + γ*_15_*(CESD_*BP*_) + γ*_16_*(Purpose × CESD) + μ*_1__*i*,_*

β*_2__*i*_* = γ_20_ + γ*_21_*(Age) + γ*_22_*(Sex) + γ*_23_*(Education) + γ*_24_*(CESD_*BP*_) + μ*_2__*i*,_*

*β_3__*i*_* = *γ*_30_ + *μ*_3__*i*,_

where the cognitive functioning (word recall or mental status) for a given person, *i*, at time, *t*, is a function of a person-specific intercept (β*_0__*i*_*), a person-specific linear slope (β*_1__*i*_*), their within-person centered level of sense of purpose and depressive symptoms at that time (Purpose_*WP*_ and CESD_*WP*_), and a residual error term (*e*_*ti*_). At the between-person level, the intercept and slope terms are expressed as overall sample means (γ*_00_* and γ*_10_*) and individual deviations around those means (μ*_0__*i*_* and μ*_1__*i*_*), as well as the effects of age, sex, education, sense of purpose, depressive symptoms, and a sense of purpose by depressive symptoms interaction term. Age and education were centered in the models on the sample mean and sex was coded as males = 0, females = 1. In addition, cross-level interaction terms on the within-person effect of sense of purpose to account for potential modification of this effect by age, sex, education, or depressive symptoms.

## Results

Baseline demographic characteristics of the sample are presented in Table 1.

**TABLE 1:** Baseline demographic characteristics (*n* = 4599).

**Variable**	**Mean (SD)/*n* (%)**	**Range**
Age	74.33 (7.11)	65–104
65–69	1430 (31.14%)	
70–79	2095 (45.55%)	
80–89	932 (20.27%)	
90+	142 (3.09%)	
Sex (female)	2614 (56.84%)	
Race		
White/Caucasian	3922 (85.28%)	
Black/African American	551 (11.98%)	
Other	126 (2.74%)	
Years of education	12.24 (3.17)	0–17
Word recall	8.98 (3.32)	0–20
Mental status	12.73 (2.49)	0–15
Depressive symptoms	1.41 (1.89)	0–8
Sense of purpose in life	4.43 (0.94)	1–6

### Word Recall

Results of the multilevel models predicting change in word recall and mental status are presented in Table 2. Having a higher sense of purpose in life predicted greater word recall at baseline, but was not associated with longitudinal change in recall across the follow-up period. However, sense of purpose did predict dynamic within-person changes in word recall. In other words, on occasions when participants reported higher purposefulness than was typical for them (i.e., above their personal mean across measurement occasions), they were more likely to perform better on the word recall memory tasks at that occasion.

**TABLE 2:** Multilevel models for sense of purpose and depressive symptoms as a predictor of word recall and mental status.

	**Word recall**		**Mental status**	
**Variable**	**Estimate (SE)**	***p***	**Estimate (SE)**	***p***
**Within-person effects**				
CESD (γ_30_)	−0.02 (0.03)	0.571	−0.01 (0.03)	0.587
Purpose (γ_20_)	0.21 (0.07)	0.001	0.13 (0.05)	0.010
Purpose × age (γ*_21_*)	0.01 (0.01)	0.068	0.02 (0.01)	<0.001
Purpose × sex (γ*_22_*)	−0.06 (0.09)	0.507	0.08 (0.06)	0.219
Purpose × education (γ*_23_*)	0.01 (0.01)	0.605	−0.01 (0.01)	0.601
Purpose × CESD (γ*_24_*)	−0.01 (0.03)	0.731	−0.00 (0.02)	0.850
**Between-person effects**				
Intercept (γ*_00_*)	8.43 (0.06)	<0.001	12.90 (0.05)	<0.001
Age (γ*_01_*)	−0.14 (0.01)	<0.001	−0.04 (0.01)	<0.001
Sex (female) (γ*_02_*)	0.97 (0.08)	<0.001	−0.34 (0.07)	<0.001
Education (γ*_03_*)	0.30 (0.01)	<0.001	0.32(0.01)	<0.001
Purpose BP (γ*_04_*)	0.36 (0.06)	<0.001	0.11(0.04)	0.011
CESD BP (γ*_05_*)	−0.17 (0.03)	<0.001	−0.14 (0.03)	<0.001
Purpose × CESD (γ*_06_*)	−0.06 (0.03)	0.041	−0.01 (0.03)	0.676
Slope (γ*_10_*)	−0.17 (0.01)	<0.001	−0.13 (0.01)	<0.001
Age (γ*_11_*)	−0.01 (0.00)	<0.001	−0.01 (0.00)	<0.001
Sex (female) (γ*_12_*)	0.00 (0.01)	0.830	−0.00 (0.01)	0.695
Education (γ*_13_*)	−0.00 (0.00)	0.206	0.00 (0.00)	0.216
Purpose BP (γ*_14_*)	0.01 (0.01)	0.396	0.01 (0.01)	0.112
CESD BP (γ*_15_*)	0.00 (0.01)	0.932	−0.01 (0.00)	0.009
Purpose × CESD (γ*_16_*)	−0.00 (0.01)	0.844	−0.00 (0.00)	0.407
**Random effects**				
Level-1 residual (*e*_*ti*_)	4.20 (0.12)	<0.001	1.73 (0.06)	<0.001
Intercept variance (μ*_0__*i*_*)	3.91 (0.15)	<0.001	3.06 (0.13)	<0.001
Slope variance (μ*_1__*i*_*)	0.01 (0.00)	<0.001	0.01(0.00)	<0.001

*CESD, number of depressive symptoms on the Center for Epidemiological Studies-Depression scale.*

**FIGURE 1:**
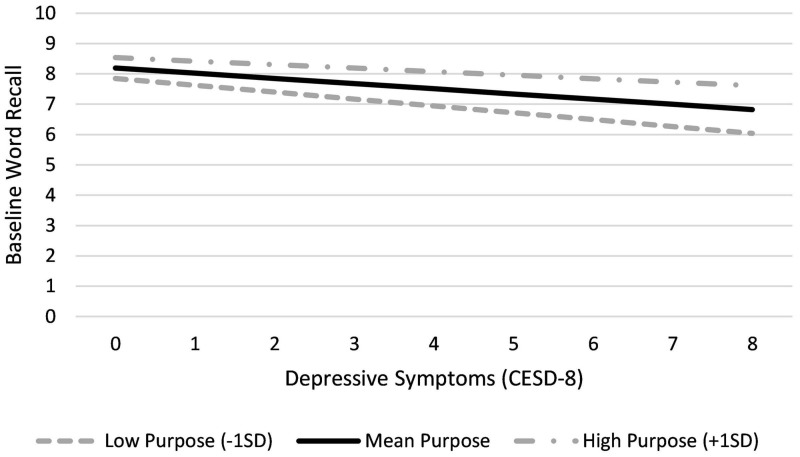
Moderating effect of sense of purpose in life on associations between depressive symptoms and baseline word recall.

Higher depressive symptoms were associated with poorer recall performance at baseline, but did not predict overall change (slope) or occasion-to-occasion fluctuations in word recall. Though higher depressive symptoms predicted poorer baseline performance, a significant sense of purpose by depressive symptoms interaction was observed wherein higher sense of purpose diminished the negative impact of depressive symptoms on word recall. For example, a participant reporting sense of purpose one standard deviation above the sample mean would be expected to recall 0.11 fewer words for each increase in depressive symptoms, whereas an individual one standard deviation below the mean for sense of purpose would recall about 0.23 fewer words with each depressive symptom. A visual representation of this interaction is presented in Figure 1.

### Mental Status

Results of the model predicting changes in the mental status composite variable are presented in the right column of Table 2. Similar to the word recall model, sense of purpose in life predicted baseline mental status, but not change in the slope parameter. Sense of purpose and performance on the mental status battery were also found to fluctuate together over measurement occasions, such that participants were likely to show concurrent increases in both sense of purpose and mental status. This within-person effect of sense of purpose on mental status was modified by age, with sense of purpose having a larger effect among older participants. Unlike the word recall model, having higher depressive symptoms was associated with both poorer initial mental status and with slightly steeper decline over time. No significant interaction was observed between sense of purpose and depressive symptoms on mental status.

## Discussion

Accruing research has supported the notion that having a sense of purpose in life is associated with positive cognitive outcomes in older adulthood including better cognitive performance (Windsor et al., 2015; Lewis et al., 2017) and resilience to dementia-related neuropathology (Boyle et al., 2012). The present study sought to build upon these past findings by examining whether sense of purpose protected against depression-related cognitive deficits in a large, representative sample of community-based American older adults. Having higher sense of purpose in life was associated with better baseline word recall and mental status, and within-person increases in sense of purpose over time predicted corresponding increases in performance on both composite cognitive measures. Moreover, higher sense of purpose in life appeared to mitigate some of the negative effects of depressive symptoms on memory performance, with the association between higher depressive symptoms and baseline word recall being weaker among participants reporting higher sense of purpose. These findings underscore the importance of having a sense of purpose in life as a source of psychological and cognitive resilience in older adulthood.

Past research has pointed to sense of purpose in life as a resource for psychological resilience, helping individuals to overcome or recover quickly in the face of challenges to health and wellbeing (Ryff et al., 2012). For instance, veterans with a history of trauma who report higher sense of purpose in life are less likely to experience psychological distress including post-traumatic stress disorder and depressive symptoms (Pietrzak and Cook, 2013). One potential explanation for sense of purpose protecting against psychological distress is reduced reactivity to stressful experiences. More purposeful individuals also appear to be less reactive to daily stressors, showing smaller increases in negative affect and physical symptoms such as headache or fatigue relative to those lower in sense of purpose (Hill et al., 2018). Purposeful individuals may also utilize more adaptive coping mechanisms to manage psychological distress, such as relying on social support or reframing challenges in the context of major life aims.

The present work also adds to a growing body of research suggesting that sense of purpose in life contributes to cognitive reserve. The theory of cognitive reserve suggests that the accumulation of certain lifestyle characteristics allow some individuals to maintain cognitive functioning in the face of neurobiological insults, such as those associated with Alzheimer’s disease and related dementias (Stern et al., 2019). In this way, cognitive reserve may be thought of as a distinction between pathology-related neural integrity and the clinical manifestations of the pathology. Indeed, post-mortem analyses have found that as many as one third of older adults with minimal cognitive impairment prior to death show levels of amyloid and neurofibrillary tangle deposition similar to persons with Alzheimer’s disease (Neuropathology Group. Medical Research Council Cognitive Function and Aging Study, 2001). Prior research has found older adults higher in sense of purpose in life to be more resilient to neuropathological changes; showing better cognitive functioning despite accruing neural hallmarks of Alzheimer’s disease relative to those lower in sense of purpose (Boyle et al., 2012). Major depressive disorder and high depressive symptomatology may similarly represent a neurological challenge insofar as it may lead to neurocircuitry abnormalities and dysregulation of neural systems involved in memory, executive functions, and attention (Murrough et al., 2011). The present study extends these findings, demonstrating that sense of purpose mitigates negative effects of depressive symptoms on memory performance.

Purpose in life may contribute to cognitive reserve through the promotion of higher order cognitive processes involved in the pursuit of purpose-driven life aims. Having a sense of purpose and direction in life is thought to involve several complex cognitive processes including self-reflection, envisioning future self-representations, and efficient allocation of resources across sometimes competing purpose-related goals (McKnight and Kashdan, 2009). Moreover, sense of purpose in life is believed to provide motivation and guide behavior toward actions that are congruent with long-term goals (Lewis, 2020). Indeed, sense of purpose is associated with greater leisure activity engagement, including participation in intellectual activities such as reading or doing puzzles (Lewis and Hill, 2020), which have been previously shown to contribute to cognitive reserve (Scarmeas and Stern, 2003). However, additional work is needed to clarify the mechanisms underlying associations between sense of purpose and higher cognitive reserve.

These findings also highlight the utility of sense of purpose in life as a therapeutic target among older adults at risk for depression and cognitive impairments. Several studies have shown that sense of purpose in life may be modifiable through brief interventions (Pizzolato et al., 2011; Chippendale and Boltz, 2015). For instance, one 8-week intervention involving training to help older adults identify and appreciate positive experiences as they relate to their purpose in life has been found to improve sense of purpose and lead to decreases in depressive symptoms, with these benefits being sustained at 6 month follow-up (Friedman et al., 2017, 2019). Such non-pharmaceutical interventions could help supplement existing treatment protocols to support wellbeing and promote cognitive resilience among older persons with depression.

The present study is limited in some ways which should serve to inform future research in the area. For instance, though the 8-item CESD scale has been shown to be a valid indicator of depressive symptomatology in large longitudinal studies (e.g., Karim et al., 2015), a detailed clinical evaluation could better clarify the role of sense of purpose in life on cognitive performance among individuals with major depressive disorder. Furthermore, the cognitive battery used in the present study was limited to two cognitive domains, which does not account for interactions between depressive symptoms and sense of purpose on other cognitive functions. Given that depression appears to be most strongly linked with deficits in tasks of attention and cognitive flexibility (Lee et al., 2012), future research is needed to ascertain whether sense of purpose similarly protects against deficits in these cognitive domains. These limitations aside, the present study highlights the value of having a sense of purpose in life as a source resilience, allowing older individuals to better maintain functioning in the face of challenges to wellbeing and cognition.

## Data Availability Statement

Publicly available datasets were analyzed in this study. This data can be found here: https://hrs.isr.umich.edu/data-products.

## Ethics Statement

The studies involving human participants were reviewed and approved by the University of Michigan. The patients/participants provided their written informed consent to participate in this study.

## Author Contributions

NL was responsible for the acquisition and analysis of the data. Both authors made substantial contributions to the conception and design of the study, as well as drafting and final approval of the manuscript.

## Conflict of Interest

The authors declare that the research was conducted in the absence of any commercial or financial relationships that could be construed as a potential conflict of interest.

## References

[B1] Boyle P. A. Buchman A. S. Barnes L. L. Bennett D. A. (2010). Effect of a purpose in life on risk of incident Alzheimer disease and mild cognitive impairment in community-dwelling older persons. *Arch. Gen. Psychiatry* 67 304–310. 10.1001/archgenpsychiatry.2009.208 20194831 PMC2897172

[B2] Boyle P. A. Buchman A. S. Wilson R. S. Yu L. Schneider J. A. Bennett D. A. (2012). Effect of purpose in life on the relation between Alzheimer disease pathologic changes on cognitive function in advanced age. *Arch. Gen. Psychiatry* 69 499–505. 10.1001/archgenpsychiatry.2011.1487 22566582 PMC3389510

[B3] Bronk K. C. Hill P. L. Lapsley D. K. Talib T. L. Finch H. (2009). Purpose, hope, and life satisfaction in three age groups. *J. Posit. Psychol.* 4 500–510. 10.1080/17439760903271439

[B4] Burrow A. L. O’Dell A. C. Hill P. L. (2010). Profiles of a developmental asset: youth purpose as a context for hope and well-being. *J. Youth Adolesc.* 39 1265–1273. 10.1007/s10964-009-9481-1 19937095

[B5] Chippendale T. Boltz M. (2015). living legends: effectiveness of a program to enhance sense of purpose and meaning in life among community-dwelling older adults. *Am. J. Occup. Ther.* 69 6904270010p1-6904270010p11. 10.5014/ajot.2015.014894 26114464

[B6] Chodosh J. Kado D. M. Seeman T. E. Karlamangla A. S. (2007). Depressive symptoms as a predictor of cognitive decline: macArthur studies of successful aging. *Am. J. Geriatr. Psychiatry* 15 406–415. 10.1097/01.JGP.0b013e31802c0c63 17353297

[B7] Dewitte L. Lewis N. A. Payne B. R. Turiano N. A. Hill P. L. (2020). Cross-lagged relationships between sense of purpose in life, memory performance, and subjective memory beliefs in adulthood over a 9-year interval. *Aging Ment. Health* 1–10. 10.1080/13607863.2020.1822284 [Epub ahead of print]. 32954859

[B8] Dickinson W. J. Potter G. G. Hybels C. F. McQuoid D. R. Steffens D. C. (2011). Change in stress and social support as predictors of cognitive decline in older adults with and without depression. *Int. J. Geriatr. Psychiatry* 26 1267–1274. 10.1002/gps.2676 21370277 PMC3280427

[B9] Dotson V. M. Resnick S. M. Zonderman A. B. (2008). Differential association of concurrent, baseline, and average depressive symptoms with cognitive decline in older adults. *Am. J. Geriatr. Psychiatry* 16 318–330. 10.1097/JGP.0b013e3181662a9c 18378557 PMC2405887

[B10] Friedman E. M. Ruini C. Foy C. R. Jaros L. Love G. Ryff C. D. (2019). Lighten UP! A community-based group intervention to promote eudaimonic well-being in older adults: a multi-site replication with 6 month follow-up. *Clin. Gerontol.* 42 387–397. 10.1080/07317115.2019.1574944 30767628 PMC6715420

[B11] Friedman E. M. Ruini C. Foy R. Jaros L. Sampson H. Ryff C. D. (2017). Lighten UP! A community-based group intervention to promote psychological well-being in older adults. *Aging Ment. Health* 21 199–205. 10.1080/13607863.2015.1093605 26460594 PMC5636191

[B12] Ganguli M. Du Y. Dodge H. H. Ratcliff G. G. Chang C.-C. H. (2006). Depressive symptoms and cognitive decline in late life: a prospective epidemiological study. *Arch. Gen. Psychiatry* 63 153–160. 10.1001/archpsyc.63.2.153 16461857

[B13] Hill P. L. Sin N. L. Turiano N. A. Burrow A. L. Almeida D. M. (2018). Sense of purpose moderates the associations between daily stressors and daily well-being. *Ann. Behav. Med.* 52 724–729. 10.1093/abm/kax039 30010709 PMC6052784

[B14] Hoffman L. Stawski R. S. (2009). Persons as contexts: evaluating between-person and within-person effects in longitudinal analysis. *Res. Hum. Dev.* 6 97–120. 10.1080/15427600902911189

[B15] Karim J. Weisz R. Bibi Z. Rehman S. U. (2015). Validation of the eight-item center for epidemiologic studies depression scale (CES-D) among older adults. *Curr. Psychol.* 34 681–692. 10.1007/s12144-014-9281-y

[B16] Kashdan T. B. McKnight P. E. (2013). Commitment to a purpose in life: an antidote to the suffering by individuals with social anxiety disorder. *Emotion* 13 1150–1159. 10.1037/a0033278 23795592 PMC4145806

[B17] Kim E. S. Hershner S. D. Strecher V. J. (2015). Purpose in life and incidence of sleep disturbances. *J. Behav. Med.* 38 590–597. 10.1007/s10865-015-9635-4 25822118

[B18] Lee R. S. C. Hermens D. F. Porter M. A. Redoblado-Hodge M. A. (2012). A meta-analysis of cognitive deficits in first-episode major depressive disorder. *J. Affect. Disord.* 140 113–124. 10.1016/j.jad.2011.10.023 22088608

[B19] Lewis N. A. (2020). Purpose in life as a guiding framework for goal engagement and motivation. *Soc. Personal. Psychol. Compass* 14:e12567. 10.1111/spc3.12567

[B20] Lewis N. A. Hill P. L. (2020). Does being active mean being purposeful in older adulthood? Examining the moderating role of retirement. *Psychol. Aging* 35 1050–1057. 10.1037/pag0000568 32790458

[B21] Lewis N. A. Turiano N. A. Payne B. R. Hill P. L. (2017). Purpose in life and cognitive functioning in adulthood. *Neuropsychol. Dev. Cogn. B Aging Neuropsychol. Cogn.* 24 662–671. 10.1080/13825585.2016.1251549 27819520

[B22] McArdle J. J. Fisher G. G. Kadlec K. M. (2007). Latent variable analyses of age trends of cognition in the health and retirement study, 1992-2004. *Psychol. Aging* 22 525–545. 10.1037/0882-7974.22.3.525 17874952

[B23] McKnight P. E. Kashdan T. B. (2009). Purpose in life as a system that creates and sustains health and well-being: an integrative. Testable Theory. *Rev. Gen. Psychol.* 13 242–251. 10.1037/a0017152

[B24] Murrough J. W. Iacoviello B. Neumeister A. Charney D. S. Iosifescu D. V. (2011). Cognitive dysfunction in depression: neurocircuitry and new therapeutic strategies. *Neurobiol. Learn. Mem.* 96 553–563. 10.1016/j.nlm.2011.06.006 21704176

[B25] Neuropathology Group. Medical Research Council Cognitive Function and Aging Study (2001). Pathological correlates of late-onset dementia in a multicentre, community-based population in England and Wales. *Lancet* 357 169–175. 10.1016/S0140-6736(00)03589-3 11213093

[B26] Nygren B. Aléx L. Jonsén E. Gustafson Y. Norberg A. Lundman B. (2005). Resilience, sense of coherence, purpose in life and self-transcendence in relation to perceived physical and mental health among the oldest old. *Aging Ment. Health* 9 354–362. 10.1080/1360500114415 16019292

[B27] Ownby R. L. Crocco E. Acevedo A. John V. Loewenstein D. (2006). Depression and risk for Alzheimer disease: systematic review, meta-analysis, and metaregression analysis. *Arch. Gen. Psychiatry* 63 530–538. 10.1001/archpsyc.63.5.530 16651510 PMC3530614

[B28] Pfund G. Hill P. (2018). The multifaceted benefits of purpose in life. *Int. Forum Logother.* 41 27–37.

[B29] Pfund G. N. Lewis N. A. (2020). “Aging with purpose: developmental changes and benefits of purpose in life throughout the lifespan,” in *Personality and Healthy Aging in Adulthood: New Directions and Techniques International Perspectives on Aging*, eds Hill P. L. Allemand M. (Cham: Springer International Publishing), 27–42. 10.1007/978-3-030-32053-9_3

[B30] Pietrzak R. H. Cook J. M. (2013). Psychological resilience in older U.S. veterans: results from the national health and resilience in veterans study. *Depress. Anxiety* 30 432–443. 10.1002/da.22083 23468170

[B31] Pizzolato J. E. Brown E. L. Kanny M. A. (2011). Purpose plus: supporting youth purpose, control, and academic achievement. *New Dir. Youth Dev.* 2011 75–88. 10.1002/yd.429 22275280

[B32] Radloff L. S. (1977). The CES-D scale: a self-report depression scale for research in the general population. *Appl. Psychol. Meas.* 1 385–401. 10.1177/014662167700100306 26918431

[B33] Raudenbush S. W. Bryk A. S. (2002). *Hierarchical Linear Models: Applications and Data Analysis Methods.* Thousand Oaks, CA: SAGE.

[B34] Rowe J. W. Kahn R. L. (1997). Successful aging. *Gerontologist* 37 433–440. 10.1093/geront/37.4.433 9279031

[B35] Ryff C. D. (1989). Happiness is everything, or is it? Explorations on the meaning of psychological well-being. *J. Pers. Soc. Psychol.* 57 1069–1081. 10.1037/0022-3514.57.6.1069

[B36] Ryff C. D. (2014). Psychological well-being revisited: advances in the science and practice of eudaimonia. *Psychother. Psychosom.* 83 10–28. 10.1159/000353263 24281296 PMC4241300

[B37] Ryff C. D. Friedman E. M. Morozink J. A. Tsenkova V. (2012). “Psychological resilience in adulthood and later life: implications for health,” in *Emerging Perspectives on Resilience in Adulthood and Later Life*, Vol. 32 eds Hayslip B. Jr. Smith G. C. (New York, NY: Springer Publishing Company), 73–92.

[B38] Scarmeas N. Stern Y. (2003). Cognitive reserve and lifestyle. *J. Clin. Exp. Neuropsychol.* 25 625–633. 10.1076/jcen.25.5.625.14576 12815500 PMC3024591

[B39] Scheier M. F. Wrosch C. Baum A. Cohen S. Martire L. M. Matthews K. A. (2006). The life engagement test: assessing purpose in life. *J. Behav. Med.* 29 291–298. 10.1007/s10865-005-9044-1 16565785

[B40] Smith G. E. (2016). Healthy cognitive aging and dementia prevention. *Am. Psychol.* 71 268–275. 10.1037/a0040250 27159433

[B41] Smith J. Ryan L. Fisher G. G. Sonnega A. Weir D. (2017). *HRS Psychosocial and Lifestyle Questionnaire 2006–2016.* Ann Arbor, MI: Survey Research Center, Institute for Social Research, University of Michigan.

[B42] Sonnega A. Faul J. D. Ofstedal M. B. Langa K. M. Phillips J. W. Weir D. R. (2014). Cohort profile: the health and retirement study (HRS). *Int. J. Epidemiol.* 43 576–585. 10.1093/ije/dyu067 24671021 PMC3997380

[B43] Stern Y. Barnes C. A. Grady C. Jones R. N. Raz N. (2019). Brain reserve, cognitive reserve, compensation, and maintenance: operationalization, validity, and mechanisms of cognitive resilience. *Neurobiol. Aging* 83 124–129. 10.1016/j.neurobiolaging.2019.03.022 31732015 PMC6859943

[B44] Weston S. J. Lewis N. A. Hill P. L. (2020). Building sense of purpose in older adulthood: examining the role of supportive relationships. *J. Posit. Psychol.* 16 1–9. 10.1080/17439760.2020.1725607

[B45] Wilson R. S. Barnes L. L. Leon C. F. M. de Aggarwal N. T. Schneider J. S. Bach J. (2002). Depressive symptoms, cognitive decline, and risk of AD in older persons. *Neurology* 59 364–370. 10.1212/WNL.59.3.364 12177369

[B46] Wilson R. S. Mendes de Leon C. F. Bienias J. L. Evans D. A. Bennett D. A. (2004). Personality and mortality in old age. *J. Gerontol. B Psychol. Sci. Soc. Sci.* 59 110–116. 10.1093/geronb/59.3.P110 15118013

[B47] Windsor T. D. Curtis R. G. Luszcz M. A. (2015). Sense of purpose as a psychological resource for aging well. *Dev. Psychol.* 51 975–986. 10.1037/dev0000023 26010384

[B48] Yaffe K. Blackwell T. Gore R. Sands L. Reus V. Browner W. S. (1999). Depressive symptoms and cognitive decline in nondemented elderly women: a prospective study. *Arch. Gen. Psychiatry* 56 425–430. 10.1001/archpsyc.56.5.425 10232297

